# Comprehensive Analysis of Brewed Tea: Boron Content, Total Antioxidant and Oxidant Capacity, Oxidative Stress, and pH value

**DOI:** 10.1007/s12011-025-04705-y

**Published:** 2025-06-18

**Authors:** Aslihan Cihan, Armağan Begum Ozel Korlu, Burcin Alev Tuzuner, Aysen Yarat

**Affiliations:** 1 Department of Biochemistry, Faculty of Pharmacy, İbrahim Çecen University, Agri, Türkiye; 2 Department of Medical Biochemistry, Faculty of Medicine, Istanbul Health and Technology University, Istanbul, Türkiye; 3 Department of Biochemistry, Doctoral Student, Faculty of Pharmacy, Institute of Health Sciences, Marmara University, Istanbul, Türkiye; 4 Department of Basic Medical Sciences, Biochemistry, Faculty of Dentistry, Istanbul Gelisim University, Avcilar Istanbul, Türkiye; 5 Life Sciences and Biomedical Engineering Application and Research Centre, Istanbul Gelisim University, Istanbul, Türkiye; 6 Department of Basic Medical Sciences, Biochemistry, Faculty of Dentistry, Marmara University, Maltepe Istanbul, Türkiye

**Keywords:** Acidity Level, Antioxidant effect, Boron, Health effect, Plant-Based-Beverage, Tea

## Abstract

Tea is widely recognized as the most consumed beverage in the world after water. In recent years, the connection between tea and health has gained growing attention as a significant research topic. In this study, we evaluated the relationship between tea and health by analyzing the boron content, total antioxidant capacity (TAC), total oxidant capacity (TOC), oxidative stress index (OSI), and pH levels of teas commonly consumed in Türkiye. A total of 42 samples were created from various tea brands and different tea types that are widely consumed in Türkiye. The determination of boron in pulverized and brewed tea samples was carried out using the carminic acid method. Additionally, boron transfer was determined in brewed teas. Brewed tea samples were also examined in terms of pH, TAC, TOC, and OSI. It was determined that all teas contained an average of 11.73 mg/L boron, the amount of boron in domestic teas was lower than in imported teas. It was determined that boron transfer into brewed tea was approximately 30%. The pH values ranged between 4–5. No differences were detected in terms of TAC, TOC, and OSI. Our research shows that tea consumption is safe in terms of boron levels and helps meet daily needs. The OSI of teas is less than 1, so their antioxidant effects can be considered quite good. However, it should be noted that excessive tea consumption can lead to the demineralization of tooth enamel due to low pH levels.

## Introduction

Tea, produced from the buds and leaves of the *Camellia sinensis* L. plant by various methods, is the most consumed beverage after water [1]. Green, black, and oolong teas represent the main categories of tea, distinguished by varying degrees of fermentation, which influence their appearance, chemical composition, taste and aroma [1, 2]. Global tea production reached 6.7 million tonnes and consumption reached 6.5 million tonnes in 2022 [3].Türkiye is the fourth-largest tea producer globally and holds the top position in per capita tea consumption, averaging 4 kg per person [4]. In addition to its sensory and health-related appeal, tea holds a prominent place in Turkish society as a traditional beverage that promotes social interaction, hospitality, and daily rituals [5].

Tea has been widely studied for its potential health benefits, primarily due to its bioactive components, such as polyphenols, amino acids, polysaccharides, alkaloids, minerals, trace elements, and vitamins [6]. The chemical ingredients of tea leaves are influenced by genetic factors, climate conditions, soil characteristics, and other environmental variables. Moreover, alterations in the biochemical composition of tea can result from the methods and standards applied during the harvesting and processing stages [4]. The probabilistic health risk assesment and concentration evaluation of various polycyclic aromatic hydrocarbons in commercial tea and coffee samples showed that the detected values were lower than the standard levels established by the European Union, and according to the Monte Carlo simulation results, the non-carcinogenic health risk for consumers was negligible. It is recommended that the processing conditions of these products be strictly controlled to prevent the formation of polycyclic aromatic hydrocarbons, due to concerns about their potential carcinogenicity and mutagenicity [7]. Tea’s bioactive substances are known to influence various physiological and biochemical pathways, such as energy metabolism, oxidative stress, inflammatory responses, and vascular function [6]. Studies suggests that drinking tea regularly is associated with a reduced risk of various chronic diseases, including diabetes [8], cardiovascular disorders [9, 10], and certain types of cancer [11], largely attributable to its potent antioxidant activity. Oxidative stress occurs when the balance between free radicals and antioxidants in the human body is disrupted. Enhancing antioxidant levels can help lower the risk of oxidative stress, as antioxidants serve to counteract the formation of harmful free radicals. Consuming foods rich in antioxidants, like tea, is considered an effective approach to reducing the risk of various complex diseases [12]. The antioxidant components of tea protect against oxidative stress and reduce the damage caused by reactive oxygen species to lipid membranes, proteins, and nucleic acids [13, 14]. The measurement of total antioxidant capacity (TAC), total oxidant capacity (TOC), and oxidative stress index (OSI) provides a comprehensive evaluation of the oxidative balance in tea and is essential for understanding their potential health-promoting antioxidant properties. The measurement of TAC, TOC, and OSI offers valuable insight into the oxidative balance of tea samples and contributes to understanding their potential antioxidant-related health benefits [15–17].

Tea is also rich in minerals and trace elements, one of which is boron. The biological role of boron, which is known to be an essential micronutrient for plants, has not yet been fully explained in animals and humans. It is suggested that boron has an important role in bone development, the antioxidant defense system, mineral and hormone metabolism, wound healing, energy metabolism and the immune system [18]. With this, excessive boron levels in the human body can lead to symptoms such as nausea, vomiting, diarrhea, and lethargy [19]. It is also stated that insufficient or toxic levels of boron intake can lead to various metabolic disorders [20]. Food and drinking water, which are the main sources of boron intake, directly reflect boron exposure in humans. It has also been stated that a boron-rich diet may have positive effects on human health [21]. The World Health Organization (WHO) has defined a safe boron intake range for adults at 0.4 mg/kg of body weight [22], while the Food and Nutrition Board has set the tolerable upper intake level for adults at as 20 mg boron/day [23]. The extraction of boron during the brewing process varies and tea infusions can serve as a dependable dietary source of boron [19]. For this reason, it is important to analyze boron levels, especially in tea samples related to human nutrition. It is suggested that impurities and pollutants in tea may pose a threat to human health when consumed. Sixteen trace elements, including boron, found in different types of tea, were analyzed during the 5-min brewing process, and their potential risks to human health were evaluated. According to the risk assessment conducted with Monte Carlo simulation, it was determined that there was no significant risk associated with tea consumption under the conditions of the study [24].

Tea has long been an important part of the diet in Türkiye, where it is consumed in large quantities and holds a significant cultural and social role [5]. Despite its health benefits, excessive consumption may lead to oral health problems such as dental erosion and staining of the dentition [25, 26]. Given that extrinsic factors such as the consumption of acidic foods and beverages play a major role in the etiology of dental erosion by contributing to the degradation of dental hard tissues, evaluating the pH of tea is important for understanding its potential impact on oral health [25].

There are limited studies from Türkiye in the literature investigating the boron content [27], antioxidant activity [4, 28] and pH levels [29], and these studies have primarily focused on herbal and fruit teas. This study aims to evaluate the boron content, TAC, TOC, OSI, and pH levels in imported and domestic black, green, white, oolong, and matcha (a type of green tea) teas available on the Turkish market. This analysis is intended to provide a more comprehensive understanding of their potential health effects and support informed dietary choices, especially considering the limited data on teas sold in Türkiye. The present study offers novel insights into boron and OSI levels, contributing valuable data to the field by including tea samples representative of the Turkish market.

## Materials and Methods

### Chemicals

The chemicals used in this study were of analytical grade and were obtained from Merck (Darmstadt, Germany), Sigma-Aldrich (St. Louis, MO, USA) and Fluka (Buchs, Switzerland) companies. The CAS numbers and firms of specific chemicals: Carminic acid (CAS: 1260–17-9, Sigma-Aldrich), boric acid (CAS: 10,043–35-3, Merck), 2,2-azino-bis (3-ethylbenzothiazoline 6-sulfonic acid) (ABTS) (CAS: 30,931–67-0, Sigma-Aldrich), xylenol orange (CAS: 3618–43-7, Merck), trolox (CAS: 53,188–07-1, Sigma-Aldrich), hydrogen peroxide (CAS: 7722–84-1,Merck), glycerol (CAS: 56–81-5, Merck), o-dianisidine-hydrochloride (CAS: 20,325–40-0, Sigma-Aldrich) and ferrous ammonium sulphate (CAS: 7783–85-9, Merck).

### Tea Samples

The sample diversity and classification of teas are shown in Table 1. In this study, a total of 42 different types of tea from 13 different brands (B: B1-B13) were analyzed. The frequently consumed teas used in the study were obtained from supermarkets with high sales circulation, while different types of imported teas, selected to ensure variety, were purchased from online tea companies. The quantities to be used were separated and numbered. Then, the tea samples were blended, bagged, labeled, and stored in boxes at room temperature. Boron amounts of dry and brewed tea samples, TAC, TOC, OSI and pH values ​​of brewed tea samples were determined as described below.

**Table 1:** Classification of tea samples examined in the study

Tea Type	Tea Samples (*n* = 42)			
	Bulk Teas (*n* = 21)		Bagged Teas (*n* = 21)	
	Domestic (B1-B6) (*n* = 13)	Imported (B7-B9) (*n* = 8)	Domestic (B1-B5) (*n* = 12)	Imported (B7-B13) (*n* = 9)
Black Tea	4	2	4	7
Organic Black Tea	1		2	
Green Tea	6	2	5	1
Organic Green Tea	1	1		
White Tea		1	1	1
Oolong Tea		1		
Matcha Tea	1	1		

B: Brand

### Boron Determination in Dry and Brewed Tea Samples

Dry tea samples were prepared by weighing 0.5 g of blended-ground tea and placing into porcelain crucibles. Due to the high volatility of boron at low pH values, sodium hydroxide was added, and the samples were dried in oven at 85 °C overnight. The dried sample was then burned in an ashing furnace at 550 °C for 4 h. After cooling in a desiccator, the sample was acidified with 6 M hydrochloric acid. It was then diluted with distilled water to a certain volume and centrifuged at 4000 rpm for 10 min (Thermo Scientific Heraeus Labofuge 200 Centrifuge). The supernatant was used for the carminic acid assay. Boron content was then determined using the modified carminic acid method given below [30, 31].

Brewed tea samples were prepared by weighing 0.5 g of blended-ground tea, and brewed with 50 mL hot water for 20 min. After brewing, the liquid part was immediately decanted to separate it from the tea particles and subsequently centrifuged at 4000 rpm for 10 min. It was divided into aliquots, stored in a deep freezer at −20 ºC [32], and later used for boron determination by the modified carminic acid method [30, 31]. In the modified carminic acid method, carminic acid and concentrated sulfuric acid were used to prepare the carmine solution (0.4 mM). Boric acid was used to prepare boron standard solutions ranging from 1 to10 mg/L. All solutions were freshly prepared before use. For the assay, 10 μL of concentrated hydrochloric acid, 1 mL of concentrated sulfuric acid, and 1 mL of 0.4 mM carmine solution were added to 0.2 mL of the supernatant. After incubation at room temperature for 45 min, absorbance was measured at 585 nm wavelength in a spectrophotometer (Rayleigh-UV-1800). All measurements were performed in duplicate. This method is based on the measurement of the absorbance at 585 nm of the colored complex formed between boron and carminic acid in a sulfuric acid medium. Boron concentrations in the samples were calculated using a boron standard curve. The lowest measurable boron concentration with this method was 0.25 mg/L [30, 31].

### Determination of Total Antioxidant Capacity in Brewed Tea

This method was performed according to Erel's method and is based on the bleaching of the characteristic color of ABTS. Antioxidants present in the sample accelerate the bleaching rate in proportion to their concentration. This reaction can be monitored spectrophotometrically and the bleaching rate is inversely proportional to the TAC of the sample [15].

For the determination of TAC, 200 µL of Reagent 1 (0.4 M acetate buffer, pH 5.8) was added to both the sample blank and sample tubes. Then, 5 µL of brewed tea sample was added. Subsequently, 20 µL of Reagent 2 (10 mM ABTS in 2 mM hydrogen peroxide in 30 mM acetate buffer, pH 3.6) was added only to the sample tube. For the sample blank, the contents were mixed and the initial absorbance was measured at 660 nm. For the sample, the mixture was incubated for 5 min, after which the final absorbance was measured at 660 nm. The difference between the initial and final absorbance values was calculated. TAC values were then determined as mM Trolox using a standard calibration curve. Standards were prepared at concentrations of 0.5, 1, 2 and 3 mM by appropriately diluting a 6 mM Trolox stock solution with 30 mM phosphate buffer (pH 7.4).

### Determination of Total Oxidant Capacity in Brewed Tea

This method was performed according to Erel's method. The oxidants present in the sample oxidize the ferrous ion-*o*-dianisidine complex to ferric ion. This oxidation reaction is enhanced by glycerol molecules in the reaction medium. Ferric ion forms a colored complex with xylenol orange in an acidic medium. The color intensity, which can be measured spectrophotometrically, is related to the total amount of oxidant molecules in the sample [16].

For the determination of TOC, 225 µL of Reagent 1 (a mixture of sodium chloride, xylenol orange, sulfuric acid, and glycerol) was added to both the sample blank and sample tubes. Then, 35 µL of brewed tea sample was added. Subsequently, 11 µL of Reagent 2 (a solution containing o-dianisidine-hydrochloride, ferrous ammonium sulphate, and sulfuric acid) was added only to the sample tube. For the sample blank, the contents were mixed and the initial absorbance was measured at 560 nm. For the sample, the mixture was incubated for 3–4 min, and then the final absorbance was measured at 560 nm. The TOC value was determined by calculating the difference between the initial and final absorbance readings, and results were expressed in µM hydrogen peroxide using a standard calibration curve. Hydrogen peroxide standards were prepared at concentrations of 12.5, 25 and 50 µM.

### Oxidative Stress Index

OSI, which allows both antioxidant and oxidant activity to be evaluated together, was calculated using the formula: OSI (arbitrary unit) = [TOC (μM)/TAC (μM)]*100. An OSI value lower than 1 means that the antioxidant effect is greater [17].

### Determination of pH in Brewed Tea

The pH of all brewed tea samples was determined using pH test strips (MColorpHast™ pH 0–14, Merck).

### Statistical Analysis

Statistical analysis was performed using GraphPad Prism 9.0 (Graphpad Software, San Diego, CA, USA). All compared data are given as mean ± standard error of the mean (SEM). For comparisons between two groups, the Student’s t-test was used for normally distributed data, while the Mann–Whitney U test was used for non-normally distributed data. For multiple comparisons, analysis of variance (ANOVA) followed by Tukey’s test was used for normally distributed data, whereas Kruskal–Wallis test followed by Dunn’s test was used for non-normally distributed data, and p < 0.05 was considered statistically significant.

## Results

### Boron Levels in Teas

Based on Table 2, among the 42 dry (unbrewed) teas used in this study, the highest boron level was found as 14.52 mg/L in black organic tea bags in domestic teas, and 19.34 mg/L in black tea bags in imported teas. The lowest boron level was found as 7.77 mg/L in green tea bags in domestic teas, and mg/L in white tea bags in imported teas.

**Table 2:** Boron levels (mg/L) in dry (unbrewed) teas

	Bulk tea				Bagged tea			
	Domestic		Imported		Domestic		Imported	
	Mean ± SEM	Min–max	Mean ± SEM	Min–max	Mean ± SEM	Min–max	Mean ± SEM	Min–max
Black tea	9.53 ± 0.32	9.30–10.81	10.73 ± 0.76	9.97–11.49	10.98 ± 0.54	9.48–11.91	14.87 ± 1.71	9.86–19.34
Organic black tea	12.96*	12.96	NA	NA	13.37 ± 1.15	12.22–14.52	NA	NA
Green tea	9.88 ± 0.48	9.06–12.17	13.76 ± 2.43	11.33–16.18	10.41 ± 0.92	7.77–13.45	13.33*	13.33
Organic green tea	11.70*	11.70	14.73*	14.73	NA	NA	NA	NA
White tea	NA	NA	16.65*	16.65	14.06*	14.06	6.64*	6.64
Oolong tea	NA	NA	10.32*	10.32	NA	NA	NA	NA
Matcha tea	8.94*	8.94	12.71*	12.71	NA	NA	NA	NA

SEM: Standard error of the mean, values are given as Mean ± SEM. Sample size (n) are not included in this table, as they are provided in Table 1 in the Materials and Methods section.* SEM cannot be calculated (single data), NA: Not available

Based on Table 3, the highest boron level in brewed teas was found in green organic bulk tea (5.53 mg/L) among domestic teas and in green bulk tea (6.85 mg/L) among imported teas. The lowest boron level was found in green bulk tea (1.21 mg/L) among domestic teas and in oolong tea (0.63 mg/L) among imported teas.

**Table 3:** Boron levels (mg/L) in brewed teas

	Bulk tea				Bagged tea			
	Imported		Domestic		Imported		Imported	
	Mean ± SEM	Min–max	Mean ± SEM	Min–max	Mean ± SEM	Min–max	Mean ± SEM	Min–max
Black tea	3.23 ± 0.27	2.63–3.77	3.42 ± 1.22	2.20–4.63	3.32 ± 0.49	2.27–4.21	3.22 ± 0.33	2.26–4.57
Organic black tea	2.82*	2.82	NA	NA	2.66 ± 0.10	2.56–2.75	NA	NA
Green tea	3.48 ± 0.65	1.21**–**4.99	4.49 ± 2.37	2.12–6.85	3.31 ± 0.66	1.46–5.37	1.65*	1.65
Organic green tea	5.53*	5.53	3.72*	3.72	NA	NA	NA	NA
White tea	NA	NA	2.35*	2.35	1.92*	1.92	3.45*	3.45
Oolong tea	NA	NA	0.63*	0.63	NA	NA	NA	NA
Matcha tea	2.35*	2.35	3.41*	3.41	NA	NA	NA	NA

SEM: Standard error of the mean, values are given as Mean ± SEM. Sample sizes (*n*) are not included in this table, as they are provided in Table 1 in the Materials and Methods section. *SEM cannot be calculated (single data), NA: Not available

**Fig. 1:**
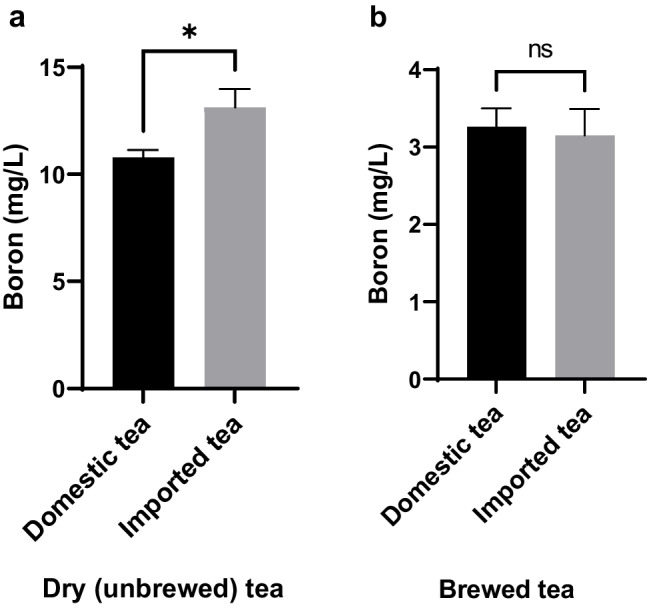
Comparison boron levels of domestic and imported dry (unbrewed) (**a**) and brewed (**b**) teas. Data are presented as mean ± SEM. *p*-values were calculated using the Mann–Whitney U test, Statistically significant changes are plotted on the graphs; *p < 0.05, ns: not statistically significant. The number of samples in each group is as follows: Domestic tea (*n* = 25), imported tea (*n* = 17)

It was found that the level of boron in dry domestic teas was significantly lower than in dry imported teas (*p* < 0.05); however, no significant difference was found between the boron levels of brewed domestic and imported teas (*p* > 0.05) (Fig. 1).

### Boron Transfer Rate to Brewed Teas (%)

When all teas were examined in terms of boron, the average boron level of dry teas decreased from 11.73 ± 0.44 mg/L to 3.22 ± 0.20 mg/L in brewed teas (*n* = 42). The ratio of boron level in dry tea to that in brewed tea was determined to be 4.40 ± 0.40. In other words, 28.99 ± 1.14% of the boron in dry teas passed into the brewed tea under the brewing conditions of this study. No significant difference was found in the boron transition percentage between domestic (31.56 ± 2.88, *n* = 25) and imported (25.21 ± 3.02, *n* = 17) brewed teas (*p* > 0.05, Mann–Whitney U).

### TAC, TOC and OSI Values ​​in Brewed Teas

The highest TAC value in brewed teas was found in black tea bag (5.88 mM) among domestic teas and in black bulk tea (5.59 mM) among imported teas. The lowest TAC value was found in green bulk tea (1.63 mM) among domestic teas and in oolong tea (0.69 mM) among imported teas (Table 4).

**Table 4:** TAC values (mM) ​​in brewed teas

	Bulk tea				Bagged tea			
	Domestic		Imported		Imported		Imported	
	Mean ± SEM	Min–max	Mean ± SEM	Min–max	Mean ± SEM	Min–max	Mean ± SEM	Min–max
Black tea	5.82 ± 0.17	4.88–5.63	4.72 ± 0.86	3.85–5.59	5.03 ± 0.34	4.29–5.88	3.34 ± 0.39	2.39–5.36
Organic black tea	4.90*	4.90	NA	NA	5.39 ± 0.21	5.17–5.60	NA	NA
Green tea	3.49 ± 0.42	1.63–4.54	4.27 ± 0.35	3.92–4.62	3.64 ± 0.43	2.80–5.25	3.80*	3.80
Organic green tea	4.36*	4.36	3.79*	3.79	NA	NA	NA	NA
White tea	NA	NA	3.56*	3.56	4.22*	4.22	4.38*	4.38
Oolong tea	NA	NA	0.69*	0.69	NA	NA	NA	NA
Matcha tea	2.05*	2.05	3.59*	3.59	NA	NA	NA	NA

SEM: Standard error of the mean, values are given as Mean ± SEM. Sample sizes (n) are not included in this table, as they are provided in Table 1 in the Materials and Methods section. *SEM cannot be calculated (single data), NA: Not available, TAC: Total antioxidant capacity

The highest TOC value in brewed teas was found in black tea bag (31.47 µM) among domestic teas and in black tea bag (41.76 µM) among imported teas. The lowest TOC value was found in green organic bulk tea (5.59 µM) among domestic teas and in green tea bag (1.47 µM) among imported teas (Table 5).

**Table 5:** TOC values (µM) in brewed teas

	Bulk tea				Bagged tea			
	Domestic		Imported		Imported		Imported	
	Mean ± SEM	Min–max	Mean ± SEM	Min–max	Mean ± SEM	Min–max	Mean ± SEM	Min–max
Black tea	17.94 ± 1.87	14.41–22.35	23.82 ± 0.00	23.82	22.35 ± 4.55	10.00–31.47	27.06 ± 0.39	15.59–41.76
Organic black tea	28.53*	28.53	NA	NA	19.42 ± 1.77	17.65–21.18	NA	NA
Green tea	15.88 ± 2.05	8.82–23.82	15.00 ± 2.35	12.65–17.35	21.29 ± 0.92	18.82–23.53	1.47*	1.47
Organic green tea	5.59*	5.59	19.41*	19.41	NA	NA	NA	NA
White tea	NA	NA	15.29*	15.29	24.71*	24.71	26.47*	26.47
Oolong tea	NA	NA	19.12*	19.12	NA	NA	NA	NA
Matcha tea	14.12*	14.12	8.53*	8.53	NA	NA	NA	NA

SEM: Standard error of the mean, values are given as Mean ± SEM. Sample sizes (n) are not included in this table, as they are provided in Table 1 in the Materials and Methods section. *SEM cannot be calculated (single data), NA: Not available, TOC: Total oxidant capacity

When the TAC values of brewed domestic and imported teas were compared, no significant difference was observed, although domestic teas had higher TAC values ​​(*p* > 0.05) (Table 6). Similarly, while the TOC values were higher in imported teas, the difference was not statistically significant (*p* > 0.05) (Table 6). In addition, no significant difference was found between domestic and imported teas in terms of OSI values (*p* > 0.05) (Table 6).

**Table 6:** TAC, TOC and OSI values in brewed teas

	Domestic tea (*n* = 25)	Imported tea (*n* = 17)	*p*
	Mean ± SEM	Mean ± SEM	
TAC (mM)	4.28 ± 0.23	4.01 ± 0.28	> 0.05^a^
TOC (µM)	18.99 ± 1.23	21.02 ± 2.36	> 0.05^b^
OSI (a.u.)	0.48 ± 0.04	0.66 ± 0.15	> 0.05^a^

^a^Mann-Whitney U test, ^b^t-test, SEM: Standard error of the mean, TAC: Total antioxidant capacity, TOC: Total oxidant capacity, OSI: Oxidative stress index

### pH Value of Brewed Teas

The pH values of all the teas examined in this study are shown in Table 7. The pH of most of the domestic and imported black tea bags and green bulk tea was around 4.5, while the pH of the others was found to be 5.

**Table 7:** pH values ​​of brewed teas

	Bulk tea				Bagged tea			
	Domestic		Imported		Domestic		Imported	
	Mean ± SEM	Min–max	Mean ± SEM	Min–max	Mean ± SEM	Min–max	Mean ± SEM	Min–max
Black tea	5.00 ± 0.00	5.00	5.00 ± 0.00	5.00	4.75 ± 0.14	4.50–5.00	4.71 ± 0.10	4.50–5.00
Organic black tea	5.00*	5.00	NA	NA	5.00 ± 0.00	5.00	NA	NA
Green tea	4.92 ± 0.08	4.50–5.00	5.00 ± 0.00	5.00	5.00 ± 0.00	5.00	5.00*	5.00
Organic green tea	5.00*	5.00	5.00*	5.00	NA	NA	NA	NA
White tea	NA	NA	5.00*	5.00	5.00*	5.00	5.00*	5.00
Oolong tea	NA	NA	5.00*	5.00	NA	NA	NA	NA
Matcha tea	5.00*	5.00	5.00*	5.00	NA	NA	NA	NA

SEM: Standard error of the mean, values are given as Mean ± SEM. Sample sizes (n) are not included in this table, as they are provided in Table 1 in the Materials and Methods section. *SEM cannot be calculated (single data), NA: Not available

Among the tea types included in the study (black, green, white, oolong, and matcha), only black and green teas had adequate sample sizes. Since there was no statistically significant difference between bagged and bulk teas, domestic and imported teas in terms of the measured parameters (*p* > 0.05) (data not shown), these forms were combined. Thus, for the purpose of comparative analysis, the samples were regrouped as black and green teas, regardless of their packaging form and origin.

**Fig. 2:**
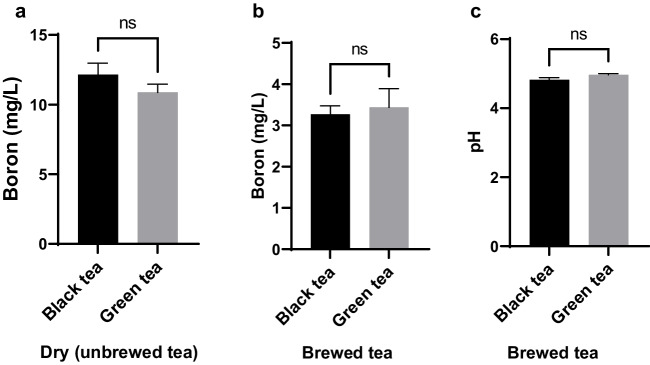
Comparison of boron levels in dry (unbrewed) black and green teas (**a**), boron levels in unbrewed black and green teas (**b**), and pH values of black and green teas (**c**). Data are presented as mean ± SEM. *p*-values were calculated using the Mann–Whitney U test for boron levels in dry teas and pH values of brewed teas; and the t-test was used for boron levels in brewed teas. Statistically significant changes are plotted on the graphs; ns: not statistically significant. The number of samples in each group is as follows: Black tea (*n* = 17); green tea (*n *= 14)

Boron levels and pH values of black and green teas are shown in Fig. 2. No statistically significant differences were observed in boron levels or pH values between black and green teas (*p* > 0.05).

**Fig. 3:**
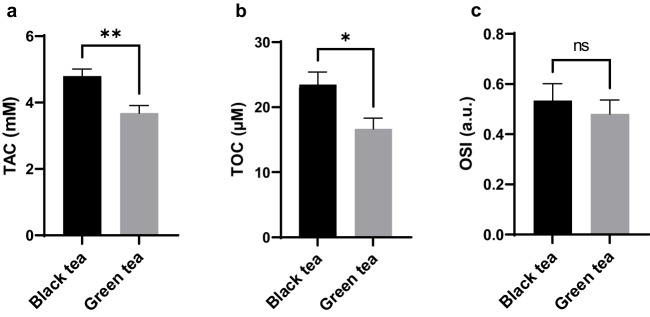
Comparison of TAC (**a**), TOC (**b**), and OSI (**c**) values between black and green brewed teas. Data are presented as mean ± SEM. a.u.:Arbitrary unit, TAC: Total antioxidant capacity, TOC: Total oxidant capacity, OSI: Oxidative stress index. *p*-values were calculated using the t-test. Statistically significant changes are plotted on the graphs; *p < 0.05, **p < 0.01, ns: not statistically significant. The number of samples in each group was as follows: Black tea (*n* = 17) and green tea (*n* = 14)

The TAC, TOC, and OSI values of black and green teas are shown in Fig. 3. The TAC and TOC values of black tea were found to be significantly higher than green tea (*p* < 0.01 and *p* < 0.05, respectively). However, the difference in OSI values between black and green teas was not statistically significant (*p* > 0.05).

## Discussion

Tea contains more than 2000 chemical components, and its composition of which varies depending on factors such as climate, soil and production methods [33]. Fresh tea leaves contain approximately 25% water, which decreases to 5% after production. The remaining content includes phenolic compounds, caffeine, proteins, amino acids, polysaccharides, carbohydrates and minerals [34]. Boron plays an important role in plant metabolism by influencing cell wall synthesis, growth, enzymatic reactions and ion transport [35]. In tea plants, boron is transported from the roots to the aerial parts as boric acid, facilitated by acidic soil and rainfall [36]. Increased soil acidity increases boron uptake, along with additional sources such as boron-containing fertilizers and pest control applications [37, 38].

Sources of boron in the human body include drinking water, tea, mineral water, milk, nuts, and exposure to natural activities (such as volcanic eruptions). Industrial products and boron-containing waste also contribute to exposure [39]. Studies have shown that boron affects various biological functions [40] and emphasize the importance of assessing boron levels in biological samples, especially in dietary sources. Although its absorption routes (ingestion, skin, inhalation) are not fully understood, boron levels in the body are estimated to range from 3–20 mg/day, with accumulation primarily in the heart (28 mg/L), bones (4.3–17.9 mg/L), and liver (2.3 mg/L). The average daily boron intake is generally 1–3 mg/day [41, 42].

Various methods are used for qualitative and quantitative boron analysis, including colorimetric, fluorimetric, volumetric, potentiometric, atomic spectrometric, chromatographic, and Inductively Coupled Plasma (ICP) techniques. ICP is highly sensitive and ideal for measuring small amounts of boron, but it is expensive. In contrast colorimetric methods using curcumin, arsenazo, or carmine are more affordable, though less sensitive than ICP [43, 44]. Carminic acid is an ideal reagent for colorimetric analysis due to its ability to minimize interference from other substances and provide reproducible, accurate results in water, plants, and soil samples [30].

In this study, the boron content of 42 tea samples (21 bulk and 21 bagged) was measured using the carminic acid method. The average boron level in domestic teas (n = 25) ranged from 7.7 to 14.45 mg/L, while that in imported teas (n = 17) ranged from 6.64 to 19.34 mg/L, with higher levels observed in the imported teas. These differences may be attributed to geographical factors. The boron levels found in this study are similar to those reported in previous investigations (3.10 to 57.8 mg/L) [45] and in a study on the micronutrient content in tea [46]. Another study on the mineral composition of tea plants reported boron levels between 10.63 and 31.58 mg/L, which is consistent with our results [47].

In our study, no significant difference was found in the level of boron between brewed domestic and imported teas. The level of boron in brewed tea samples was 3.22 mg/L. In other words, approximately 30% of the boron content in dry tea was extracted into the infusion under the brewing conditions applied in this study.

A study conducted in Poland measured boron concentrations in the water-soluble and acid-soluble fractions of black and fruit teas using inductively coupled plasma-atomic emission spectrometry to assess potential health risks. The average boron content in black teas ranged from 8.31 to 18.40 mg/kg, with extraction rates between 8 and 27%. The study concluded that there is no health risk from boron in tea unless metal-contaminated foods are consumed simultaneously [19]. The findings of this study are similar to those reported by Ziola-Frankowska, although the analytical methods differ (ICP vs carminic acid). Nevertheless, boron concentrations determined by the ICP and the carminic acid methods have been reported to be correlated [31]. Another study from Türkiye reported boron levels in brewed teas ranging from 0.084 to 2.023 mg/L after five minutes of infusion, which is lower than the levels found in our study using a 20-min infusion [27].

Factors such as age, gender and metabolic rate influence boron intake. The European Food Safety Authority recommends the following daily boron intake: 3 mg for ages 1–3, 4 mg for ages 4–6, 5 mg for ages 7–10, 7 mg for ages 11–14, 9 mg for ages 15–17 and 10 mg for adults. The WHO has defined a safe intake range for adults as 1–13 mg/day, which was later revised to 28 mg/day for a 70 kg adult. These recommended values vary across countries and organizations over time, and influence the regulatory limits set for boron levels in drinking water, tea and mineral water [22, 31, 48, 49]. In our previous study, the average daily boron intake from brewed tea was 1.75 mg, assuming an individual consumes five glasses of tea (500 mL) daily [32]. Similarly, in the present study, the average daily boron intake from brewed tea was calculated to be 1.61 mg, which remains within safe limits for consumption.

Tea is consumed for its antioxidant [50], antimutagenic, anticarcinogenic [51], antiviral, antifungal [52], antiallergic [53] and antimicrobial [54] effects. Polyphenols and flavonoids, which are commonly found in tea, fruits, vegetables, nuts, seeds, wine, and honey [55], contribute to various health benefits. The composition of tea leaves varies according to climate, production, and genetics. Green tea contains 160–1500 mg/g of flavonoids, while black tea contains 120–1300 mg/g, which is much higher than the flavonoid content of fruits such as apples and grapes [56].

Carloni et al. investigated the antioxidant activities of white, black and green teas in a study in which the teas were brewed at a concentration 2.5 g/100 g. They determined the total phenolic content of green teas as 23.6 and 22.6 mM gallic acid equivalents (GAE), and that of black teas as 10.7 and 14.9 mM GAE. They reported that antioxidant activity decreased from green to white to black tea, which was consistent with the total phenolic content. They also emphasized that the manufacturing process plays a significant role in determining tea properties [57]. The effect of brewing time and temperature of tea on the total phenolic content and antioxidant activity was revealed in a previous study [58]. The highest antioxidant activity value was determined in green tea samples (1942.761 µmol Trolox equivalent/L) [59]. Antioxidant activity generally follows the order green tea > oolong tea > black tea [57]. However, some studies suggest that black tea may exhibit higher antioxidant activity than green tea [60–62], while others report no significant difference between them [63, 64]. Another previous study reported that the antioxidant capacity composite index was highest in extracts of green tea, followed by oolong, white, and black teas. Tea polyphenols and catechin components were identified as key contributors to antioxidant activity in all tea types [65]. Moreover, one study measured the total phenolic content and antioxidant activity of various teas, including linden, fennel, sage, mate, echinacea, chamomile, jersey, black and green tea. The highest phenolic content was found in dry and brewed green and black teas, while the highest antioxidant activity in brewed teas was in linden, mate, echinacea and sage. No correlation was found between phenolic content and antioxidant activity in either dry or brewed teas [66]. This lack of correlation may be due to the synergistic or antagonistic interactions among various bioactive compounds. It is suggested that total antioxidant activity is not solely dependent on a specific type of polyphenol but rather results from the combined effects of various antioxidants, including catechins and other polyphenolic compounds [65]. In one study, the total antioxidant activity of green tea leaves was found to be 660.75 mmol-eqv./mres.dry weight using potentiometric measurement with a platinum electrode and pH meter. It has been shown that green tea leaves have high antioxidant activity and are rich in phenolic compounds, with a strong correlation observed between phytochemical content and antioxidant activity [67]. In another study, the total antioxidant capacity of four tea brands (A, B, C, D) was measured using a digital image-based colorimetric system and the values ​​of 380 ± 8 mg/L, 402 ± 4 mg/L, 213 ± 3 mg/L and 232 ± 4 mg/L were obtained as ascorbic acid equivalents, respectively [68].

The demand for organic foods has increased due to their perceived health, taste and environmental benefits [69]. While some studies support the superiority of organic foods in terms of health, nutrition and quality [70–72], others have found no significant difference [73, 74]. One important factor for comparision is antioxidant activity [75].

In a study on brewed organic teas, some organic teas were found to have higher antioxidant and oxidant capacities compared to their non-organic teas, suggesting that antioxidant/oxidant levels may vary depending on plant type, dose and brewing method. Based on the OSI value, which evaluates both antioxidant and oxidant activity together, chamomile tea was reported to have the highest OSI value (3.435) [76]. In the present study, the OSI value ​​of brewed teas was around 0.5, indicating a higher antioxidant effect.

Experimental studies have investigated the effects of tea on health [77]. Zeyuan et al. found that black tea was more effective than green tea in increasing erythrocyte antioxidants [78]. Langley-Evans reported that 35–45% of dietary antioxidants are derived from tea flavonoids and that higher brewing temperatures increase antioxidant extraction [79]. Yen et al. reported that 48% of the daily flavonoid intake (23 mg) comes from tea [56]. Vinson and Dabbagh emphasized that daily tea consumption in the United States provides 200–300 mg of flavonoids, exceeding the recommended intake levels of vitamins C, E, and β-carotene (70 mg/day) [80]. Yang and Landau showed that tea phenolics inhibit the proliferation of skin and lung tumor cells [81], while Vinson and Dabbagh and Langley-Evans found that tea delayes the oxidation of low-density lipoproteins and increases plasma antioxidant levels [80, 82].

Tea quality is influenced by various factors, including light, temperature, and brewing conditions such as temperature and duration, all of which significantly affect the final infusion quality. Elevated temperatures notably promote free radical formation. Therefore, in this study, we assessed TOC to quantify the overall oxidative load in teas. TOC is particularly valuable for evaluating the extent of oxidation and degradation of food components [83]. Various methods are used to determine total antioxidant levels, including TAC and TOC techniques [68]. In the present study, TAC was measured by bleaching ABTS; and antioxidants accelerate the bleaching rate and this rate is inversely proportional to TAC [15]. For TOC, oxidants in the sample oxidize an iron ion complex to form a colored complex with xylenol orange, and this rate is proportional to the oxidant concentration [16]. Oxidative stress index (0.5) indicates high antioxidant properties in brewed teas [17]. In the present study, no significant difference were found in TAC (~ 4 mM) and TOC (~ 20 µM) values ​​between domestic and imported brewed teas. This may be due to the use of different tea brands. Moreover, comparing black and green teas, no significant differences were found in boron levels, pH or OSI, while TAC and TOC values ​​were significantly higher in black tea, indicating greater antioxidant capacity and total oxidant content. This finding is consistent with some previous studies suggesting stronger antioxidant activity in black tea than in green tea [60–62], although other studies have reported higher TAC and lower TOC values ​​in green tea [84, 85] or found no significant difference between the two [63, 64]. Interestingly, although black tea may exhibit higher antioxidant activity by some measures, green tea generally contains more polyphenols, and the IC_50_ values ​​of both teas are inversely proportional to their polyphenol content, suggesting a relationship between polyphenol levels and toxicity [86]. In addition, the antioxidant and oxidant activities of a mixed tea prepared from 10 different herbs were tested individually and in combination and OSI values ​​were calculated, leading to the conclusion that it is not possible to generalize whether herbal or mixed teas are inherently beneficial or harmful [87]. These mixed findings reflect a broader uncertainty in the literature. Some studies report that green tea may protect against oxidative damage, while others associate high black tea consumption with increased markers of oxidative stress. Overall, the effect of tea consumption on oxidative stress is not definitive, highlighting the need for further research on its specific mechanisms and long-term effects.

Tea has also been reported to offer potential benefits for oral health. Its high fluoride content is well documented in the literature. Additionally, tannic acid has been found to suppress the growth of *Streptococcus mutans*, while certain flavonoids, particularly catechins, have demonstrated inhibitory effects on cariogenic bacteria [88]. However, recent lifestyle changes have increased the consumption of acidic products, leading to an increase in the incidence of dental erosion, especially among children and adolescents [89]. Tea, which is widely consumed in Türkiye, is generally considered a healthier alternative, but it is known to have an erosive effect on tooth enamel [90]. The erosive potential of foods and beverages depends on factors such as pH, acidity, mineral content, and calcium chelation [91, 92]. In a study conducted on herbal and fruit teas in Türkiye, the pH values ​​of fruit teas ranged from 2.72 to 3.62, whereas herbal teas had pH values between 6.47 and 7.24 at different brewing times (0, 2, 5, 10 min). Blackberry tea was found to be the most acidic (pH 2.7) [29]. A similar study conducted in Brazil showed that ready-to-drink teas had lower pH values (2.89 to 4.03) and higher acidity, indicating a higher erosive potential compared to brewed teas, which had pH values ​​above 7.0 [26]. Our study also found that most domestic and imported black tea bags have a pH value of approximately 4.5, which is below the critical pH of 5.5 required for enamel protection. Based on these findings, it is recommended to drink water after tea consumption to help raise oral pH.

One of the limitations of our study is that a greater number of tea samples and a wider range of parameters could not be evaluated due to lack of financial resources. The experiments were conducted and evaluated as much as possible within the available means. Another limitation of the study is the absence of Monte Carlo simulation for health risk assement and lack of Principal Component Analysis.

## Conclusion

This study investigated boron content, TAC, TOC, OSI, and pH levels in various teas from the Turkish market.The findings showed that boron concentrations remained within the tolerable upper intake level, with about 30% of the boron content extracted into the infusion, supporting the safety of consumption in terms of boron exposure while also contributing to dieatary requirements. This may help maintain physiological homeostasis and support the prevention or management of various diseases. The TAC of the teas was notable, with OSI values consistently below 1, indicating strong antioxidant potential. Black tea showed significantly higher TAC and TOC values compared to green tea. No significant OSI difference was observed between black and green teas. Many teas had a pH around 5, which is below the critical threshold required for enamel protection. Although teas exhibited strong antioxidant potential, excessive consumption may risk enamel demineralization. Drinking water after tea consumption is recommended to help restore oral pH.

## Data Availability

No datasets were generated or analysed during the current study.
